# Hypertrophy is induced during the *in vitro *chondrogenic differentiation of human mesenchymal stem cells by bone morphogenetic protein-2 and bone morphogenetic protein-4 gene transfer

**DOI:** 10.1186/ar2822

**Published:** 2009-10-02

**Authors:** Andre F Steinert, Benedikt Proffen, Manuela Kunz, Christian Hendrich, Steven C Ghivizzani, Ulrich Nöth, Axel Rethwilm, Jochen Eulert, Christopher H Evans

**Affiliations:** 1 Orthopaedic Center for Musculoskeletal Research, Orthopaedic Clinic, König-Ludwig-Haus, Julius-Maximilians-University, Brettreichstrasse 11, 97074 Würzburg, Germany; 2 Center for Molecular Orthopaedics, Harvard Medical School, 221 Longwood Avenue, BLI 152, Boston, MA 02115, USA; 3 Department of Orthopaedics and Rehabilitation, University of Florida, 3450 Hull Road, Gainesville, FL 32607, USA; 4 Institut für Virologie und Immunbiologie, Julius-Maximilians-University, Versbacherstrasse 7, 97078 Würzburg, Germany

## Abstract

**Introduction:**

The present study compares bone morphogenetic protein (BMP)-4 and BMP-2 gene transfer as agents of chondrogenesis and hypertrophy in human primary mesenchymal stem cells (MSCs) maintained as pellet cultures.

**Methods:**

Adenoviral vectors carrying cDNA encoding human BMP-4 (Ad.BMP-4) were constructed by cre-lox combination and compared to previously generated adenoviral vectors for BMP-2 (Ad.BMP-2), green fluorescent protein (Ad.GFP), or firefly luciferase (Ad.Luc). Cultures of human bone-marrow derived MSCs were infected with 5 × 10^2 ^viral particles/cell of Ad.BMP-2, or Ad.BMP-4, seeded into aggregates and cultured for three weeks in a defined, serum-free medium. Untransduced cells or cultures transduced with marker genes served as controls. Expression of BMP-2 and BMP-4 was determined by ELISA, and aggregates were analyzed histologically, immunohistochemically, biochemically and by RT-PCR for chondrogenesis and hypertrophy.

**Results:**

Levels of BMP-2 and BMP-4 in the media were initially 30 to 60 ng/mL and declined thereafter. BMP-4 and BMP-2 genes were equipotent inducers of chondrogenesis in primary MSCs as judged by lacuna formation, strong staining for proteoglycans and collagen type II, increased levels of GAG synthesis, and expression of mRNAs associated with the chondrocyte phenotype. However, BMP-4 modified aggregates showed a lower tendency to progress towards hypertrophy, as judged by expression of alkaline phosphatase, annexin 5, immunohistochemical staining for type X collagen protein, and lacunar size.

**Conclusions:**

BMP-2 and BMP-4 were equally effective in provoking chondrogenesis by primary human MSCs in pellet culture. However, chondrogenesis triggered by BMP-2 and BMP-4 gene transfer showed considerable evidence of hypertrophic differentiation, with, the cells resembling growth plate chondrocytes both morphologically and functionally. This suggests caution when using these candidate genes in cartilage repair.

## Introduction

Mesenchymal progenitor cells, also referred to as mesenchymal stem cells (MSCs), provide an attractive alternative to chondrocytes with regard to cell-based approaches to cartilage repair [1]. With the use of the proper three-dimensional serum-free culture conditions, expanded MSCs can be stimulated to differentiate along the chondrogenic pathway when the appropriate factors, such as certain members of the transforming growth factor (TGF)-β superfamily, are present [2-4]. This research has led to the first clinical application of autologous bone marrow stromal cells for the repair of full-thickness articular cartilage defects in humans [5,6]. However, to date, the delivery of MSCs into cartilaginous lesions has neither clinically nor experimentally resulted in sustained regeneration of hyaline cartilage *in vivo *[7]. Inadequate delivery of the soluble factors necessary to drive the chondrogenic differentiation of the transplanted cells *in vivo *is a major impediment to effective chondrogenesis *in situ *[7]. To overcome this limitation, gene transfer approaches are being explored clinically [8] and experimentally [9-12] to enable the sustained delivery of chondrogenic and anti-inflammatory factors to cartilage defects.

Another obstacle was identified from studies of *in vitro *chondrogenesis using MSCs or chondrocytes treated with bone morphogenetic proteins (BMPs), members of the TGF-β superfamily. BMPs are a group of secreted polypeptides with pleiotropic roles in many different cell types and were originally identified by their ability to induce endochondral bone formation in ectopic extraskeletal sites *in vivo *[1,7-10]. Among other BMPs, BMP-2 and BMP-7 are known to induce differentiation of mesenchymal progenitor cells and preosteoblasts into mature osteoblasts, and to enhance the differentiated function of osteoblasts, which have led to the clinical application of these proteins for bone regeneration [1,7-10]. We and others have tested several BMPs for their potential use in cartilage regeneration including BMP-2, BMP-4, BMP-6 and BMP-7, which were shown to induce chondrogenic differentiation of mesenchymal progenitor cells and to up regulate the levels of type II collagen and aggrecan in chondrocytes and chondroprogenitor cells [1,7-11]. During development of the limbs, however, BMPs along with other regulators also mediate the replacement of chondrogenesis by endochondral ossification comprising chondrocyte maturation, hypertrophy, transition from type II to type X collagen with subsequent chondrocyte apoptosis, while osteoprogenitor cells differentiate into osteoblasts and replace the cartilage with mineralized bone tissue. Equivalently, chondrogenic cultures induced by BMPs showed high expression of genes associated with chondrocyte hypertrophy, including collagen type (COL) X and indian hedgehog (IHH), among others [1,7-11,13]. This suggests that the chondrogenic differentiation of the MSCs advanced to the end stage, hypertrophic state that is typical of endochondral ossification during skeletal development. This conclusion correlates well with existing *in vivo *data. For example, delivery of BMP-2 expressing MSCs resulted in tissue hypertrophy and the formation of osteophytes, when transplanted orthotopically to osteochondral defects [14] or ectopically [15,16] in small animal models. Moreover, such hypertrophy-associated changes are not exclusively found in terminal differentiated growth plate chondrocytes, but are also present in pathological conditions such as osteoarthritis [17,18].

Inspired by these observations, we aim to further explore the effects of chondrogenic-induction by BMPs on hypertrophy, maturation and apoptosis. We have previously shown that adenoviral delivery of individual cDNAs encoding BMP-2 or TGF-β1 into primary MSCs is capable of driving chondrogenesis in culture [19,20]. In the present study, using adenoviral-mediated gene transfer our aim was to compare the effects of BMP-4 and BMP-2 expression on chondrogenesis of primary MSCs and to investigate whether levels and extent of hypertrophy *in vitro *is influenced by the choice of transgene.

## Materials and methods

### Construction and preparation of recombinant adenoviral vectors

The complete coding sequence of the human BMP-4 gene [GenBank:M22490] cloned into λ gt10 bacteriophage vectors (ATCC No. 40342; Manassas, VA, USA) was isolated and purified according to standard protocols [21]. The isolated λ gt10 DNA was then digested with EcoRI to release the 1.7 kB sized BMP-4 cDNA insert, which was then cloned into the EcoRI site of the pAdlox shuttle vector, and first-generation, E1, E3-deleted, serotype 5 adenoviral vectors carrying the cDNAs for human BMP-4 were constructed by *cre*-*lox *recombination as previously described [22]. The vectors encoding BMP-2, firefly luciferase (Luc) or green fluorescent protein (GFP) from jellyfish were generated previously [22]. The resulting vectors were designated Ad.BMP-2, Ad.BMP-4, Ad.Luc and Ad.GFP, and suspensions of recombinant adenovirus were prepared by amplification in 293 cells followed by purification using three consecutive CsCl gradients [22]. Viral titers were estimated to be between 10^12 ^and 10^13 ^particles/mL by optical density at 260 nm and standard plaque assay.

### Culture of human bone marrow-derived MSCs and adenoviral transduction

Bone marrow was harvested from the surgical waste of femurs of six patients, aged 48 to 63 years (mean age 55 years), undergoing total hip arthroplasty, after informed consent was given and as approved by the institutional review board of the University of Wuerzburg as described earlier [23]. The collected cells were spun at 1 × 10^3 ^rpm for five minutes, resuspended in complete DMEM (containing 10% fetal bovine serum (FBS) and 1% penicillin/streptomycin), and plated at 4 to 6 × 10^7 ^nucleated cells per 75 cm^2 ^flask (Falcon, Beckton Dickinson Labware, Franklin Lakes, NJ, USA). Unattached cells were removed after three days, and adherent colonies were cultured at 37°C, 5% CO_2 _in DMEM with 10% FBS supplemented with 1 ng/mL fibroblast growth factor (FGF) -2 for expansion of chondroprogenitor cells. Medium changes were performed every three to four days, and after 14 days adherent colonies were trypsinized and replated in several 75 cm^2 ^tissue culture flasks. At confluence (approximately 1.2 × 10^6 ^cells/T-75 flask), the cultures were infected in 750 μL serum-free DMEM for two hours at a dose of 5 × 10^3 ^vp/cell of Ad.BMP-2, or Ad.BMP-4. Control cultures were similarly infected with Ad.GFP or Ad.Luc at 5 × 10^3 ^vp/cell, or remained uninfected. For comparison, an additional set of untransduced recombinant human protein controls were maintained, which were cultured in the presence of 10 ng/mL TGF-β1 protein, or 25 ng/mL BMP-2, or 25 ng/mL BMP-4 (all R&D Systems, Minneapolis, MN, USA). Following viral infection, the supernatant was aspirated and replaced with 10 mL complete DMEM.

### Aggregate culture and transgene expression

Twenty-four hours post-infection, the MSC cultures were trypsinized, washed and placed in aggregate culture as described previously [24], and as modified by Penick and colleagues [25]. Briefly, MSCs were suspended to a concentration of 1 × 10^6 ^cell/mL in serum-free DMEM containing 1 mM pyruvate, 1% ITS + Premix (insulin, transferrin and selenous acid containing culture supplement), 37.5 mg/mL ascorbate-2-phosphate and 10^-7 ^M dexamethasone (all Sigma, St. Louis, MO, USA), and 200 μL aliquots (2 × 10^5 ^cells) were distributed to a polypropylene, v-bottom 96-well plate (Corning, Corning, NY, USA) to promote aggregate formation. As mentioned above, to particular control aggregates 25 ng/mL BMP-2, 25 ng/mL BMP-4, or 10 ng/mL TGF-β1 recombinant protein (all R&D Systems, Minneapolis, MN, USA) was added to induce chondrogenesis. The cell pellets were cultured at 37°C, 5% CO_2 _and formed spherical aggregates within 24 hours. Changes of media were performed every two to three days, with the recombinant protein being also freshly added to the respective controls. The aggregates were harvested at various time points for further analyses.

Media conditioned by the aggregates over a 24-hour period were collected at day 3, 7, 14 and 21 of culture and assayed for BMP-2 and BMP-4 expression using the appropriate commercially available ELISA kits (R&D Systems, Minneapolis, MN, USA).

### Cell proliferation, glycosaminoglycan and alkaline phosphatase assays

For analysis of cell proliferation in aggregates, the WST1 test was performed at day 3, 7, 14 and 21 of culture according to the directions of the supplier (Boehringer, Ingelheim, Germany). Briefly, at time points indicated, pellets were washed twice with PBS and incubated with the WST1 reagent for two hours at 37°C. After this incubation, the formazan dye produced by metabolically active cells was quantified by measuring the absorbance at 450/690 nm in 96-well plates (Falcon).

Cell proliferation in aggregates was further assessed by quantitative detection of adenosine 5'-triphosphate (ATP), which correlates with the number of viable cells present using the CellTiter-Glo^® ^Luminescent Cell Viability Assay (Promega, Mannheim, Baden-Würtemberg, Germany) according to the manufacturer's instructions. Briefly, pellets were homogenized mechanically using a pellet pestle and mixed with 100 μL of CellTiter-Glo^® ^reagent, which was generated by reconstitution of CellTiter-Glo^® ^substrate with CellTiter-Glo^® ^buffer. After incubation for 10 minutes at room temperature luminescence was measured using a plate-reading luminometer.

For analysis of glycosaminoglycan (GAG) content, aggregates were washed with PBS, digested with 200 μL of papain digest solution (1 μg/mL, Sigma, St. Louis, MO, USA), and incubated for 16 hours at 65°C. Total GAG content was measured by reaction with 1,9-dimethylmethylene blue using the Blyscan™ Sulfated Glycosaminoglycan Assay (Biocolor Ltd., Newtownabbey, Northern Ireland) as directed by the supplier. For normalization, DNA content of aggregates was also determined fluorometrically using the Quant-iT™ PicoGreen^® ^kit as directed by the supplier (Invitrogen GmbH, Karlsruhe, Germany).

Alkaline phosphatase (ALP) activity was measured densitometrically using change in absorbance at 405 nm by the conversion of p-nitrophenyl phosphate to p-nitrophenol and inorganic phosphate, as described previously [26]. Briefly, aggregates were homogenized mechanically and incubated with 0.1 mL of alkaline lysis buffer (0.1 M glycin, 1% triton X-100, 1 mM MgCl_2_, 1 mM ZnCl_2_) at room temperature for one hour. Thereafter 100 μL of lysis buffer was added which was supplemented with p-nitrophenylphosphate (2 mg/mL; Sigma, St. Louis, MO, USA), and stopped after 15 minutes with 50 μL 50 mM NaOH before optical densities were determined at 405 nm in an ELISA reader. ALP activity was referred to a standard curve made from p-nitrophenol (Sigma, St. Louis, MO, USA), and normalized to the DNA content and given as relative ALP activity in U/μg.

### Histological and immunohistochemical analyses

For histological analyses, aggregates were fixed in 4% paraformaldehyde for one hour before tissue processing. After dehydration in graded alcohols, the aggregates were paraffin embedded, and sectioned to 5 μm. Representative sections were stained using H&E for evaluation of cellularity and alcian blue (Sigma, St. Louis, MO, USA) for the detection of matrix proteoglycan. ALP activity was also detected by a histochemical assay performed according to the manufacturer's protocol (Sigma, St. Louis, MO, USA) and alternate sections were used for immunohistochemistry.

For immunohistochemical analyses, sections were washed for 20 minutes in Tris-buffered saline (TBS), and incubated in 5% BSA (Sigma, St. Louis, MO, USA). Following washing in TBS, sections were pre-digested with pepsin at 1 mg/mL in Tris-HCl (pH 2.0) for 15 minutes at room temperature for COL II detection, or with chondroitinase ABC (Sigma, St. Louis, MO, USA) for 10 minutes for chondroitin-4-sulfate (CS4) detection (5 U/mL in distilled water), or with 0.25% trypsin containing 1 mM EDTA for 15 minutes at 37°C for COL X detection, before sections were incubated overnight at 4°C primary antibodies diluted in 0.5% BSA. As primary antibodies monoclonal anti-COL II (Acris Antibodies GmbH, Hiddenhausen, Germany), anti-CS4 (Millipore GmbH, Schwalbach, Germany) or anti-COL X antibodies (Calbiochem, Bad Soden, Germany) were used. Immunostaining was visualized by treatment with peroxidase-conjugated antibodies (Dako, Hamburg, Germany) followed by diaminobenzidine staining (DAB kit; Sigma, St. Louis, MO, USA). The slides were finally counterstained with hemalaun (Merck, Darmstadt, Germany). For all immunohistochemical analyses, controls with non-immune immunoglobulin (Ig) G (Sigma, St. Louis, MO, USA) instead of the primary antibodies were performed.

Although more sophisticated and accurate methods of lacunae size determination have been described [27], we used a simple random field histomorphometric cell surface area measurement procedure to approximate cell sizes in aggregates. For each aggregate analyzed, three individual mid-sections stained with H&E or alcian blue were taken, and the surface areas of 10 randomly chosen lacunae by two independent investigators (AFS and BP) in a blinded fashion were measured from each of three representative microscope views taken from the center or the periphery (outer 200 μm area) section using the KS 400^® ^computerized image analysis system (Carl Zeiss GmbH, Jena, Germany). At least three different aggregates per group and bone marrow preparations from five different preparations were analyzed.

For comparison, we also analyzed the sizes of the lacunae within different zones of growth plate cartilage obtained from a four-year-old child, from whom a sixth toe was removed. Specifically, from the toe we obtained four physes (two joints) and at least three sections per physis were analyzed by measuring the surface areas of 10 randomly chosen lacunae from each of three representative microscope views by two independent investigators (AFS and BP). The lacunae were taken from the reserve, proliferative, hypertrophic or calcifying zone.

### Cell viability and apoptosis assay

As annexin 5 (Ann5) is expressed by hypertrophic chondrocytes and in osteoarthritic cartilage [17], we were next interested in the appearance of live and apoptotic cells within our aggregate system after 10 and 21 days, which was visualized using the Ann5-Cy3 apoptosis detection kit (APOAC; Sigma, St. Louis, MO, USA) as directed by the supplier. The assay uses the Cy3.18 dye as red fluorochome conjugated with Ann5-Cy3 for apoptosis detection through binding to phosphatidylserine epitopes on the plasma membrane of early apoptotic cells, and the hydrolysis of the non-fluorescent 6-carboxyfluorescein diacetate (6-CFDA) to the green fluorescent compound 6-carboxyfluorescein by the esterases of living cells to label viable cells. This combination allows the differentiation among early apoptotic cells (Ann5 positive, 6-CFDA positive), necrotic cells (Ann5 positive, 6-CFDA negative), and viable cells (Ann5 negative, 6-CFDA positive). Aggregates were incubated with 50 μL of the double labelling staining solution for 10 minutes at room temperature. After staining, aggregates were washed five times with 100 μL of binding buffer, fixed overnight in PBS-buffered 4% paraformaldehyde, dehydrated, infiltrated with isoamylacetate (Merck, Hohenbrunn, Germany), embedded in paraffin, and sectioned to 4 μm. Viable and non-viable cells were observed on the respective mid-sections using a fluorescence microscope and the appropriate green and red filters.

### Total RNA extraction, semi-quantitative and real-time RT-PCR

RNA was extracted from MSC aggregates at the indicated time-points. For this, 6 to 10 pellets per group and time point for each donor were pooled and homogenized using a pellet pestle and repeated titration in 1 mL of Trizol reagent (Invitrogen, Karlsruhe, Germany). Total RNA was subsequently extracted using Trizol reagent with an additional purification step using separation columns (NucleoSpin RNA II kit; Macherey-Nagel GmbH, Düren, Germany) including a DNase treatment step according to the manufacturer's instructions. RNA from aggregates of each condition (2 μg each group) was used for random hexamer primed cDNA synthesis using BioScript reverse transcriptase (Bioline GmbH, Luckenwalde, Germany).

For semi-quantitative PCR analyses equal amounts (100 ng) of each cDNA were used as templates for amplification in a 30 μL reaction volume using MangoTaq DNA Polymerase Taq (Bioline GmbH, Luckenwalde, Germany) and 5 pmol of gene-specific primers, which were used to detect mRNA transcripts characteristic of chondrogenic, hypertrophic or osteogenic differentiation states. The sequences, annealing temperatures and product sizes of forward and reverse primers used for COL II, aggrecan core protein (AGC), cartilage oligomeric matrix protein (COMP), fibromodulin (FMD), SRY (sex determining region Y) - box9 (SOX9), COL I, COL X, osteopontin (OP), IHH, runt-related transcription factor 2 (RUNX 2) are listed in Table 1, with elongation factor 1α (EF1α) serving as housekeeping gene and internal control. The RT-PCR products were electrophoretically separated on 1.5% agarose gels containing 0.1 mg/mL ethidium bromide and visualized using the Bio Profile software (LTF, Wasserburg, Germany), allowing correlation between EF1α signals and cycle number for each sample. The densities of the PCR bands were analyzed with the Bio 1D/Capt MW software (LTF, Wasserburg, Germany) and the mean ratio (fold change), normalized to expression of the EF1α housekeeping gene, was calculated from three bands (one per patient).

**Table 1:** Primer sequences and product sizes, for semi-quantitative and real-time RT-PCR

Gene	RT-PCR primer sequences (5'-3')	Annealing temp. (°C)	Product size (bp)	Cycles
**Chondrogenic markers**				
COL II	Sense: TTTCCCAGGTCAAGATGGTC Antisense: CTTCAGCACCTGTC CACCA	58	374	35
AGC	Sense: TGAGGAGGGCTGGAACAAGTACC Antisense: GGAGGTGGTAATTGCAGGGAACA	54	392	30
COMP	Sense: CAGGACGACTTTGATGCAGA Antisense: AAGCTGGAGCTGTCTGGTA	54	312	32
FMD	Sense: CTTACCCCTATGGGGTGGAT Antisense: GTACATGGCCGTGAGGAAGT	54	389	35
SOX9	Sense: ATCTGAAGAAGGAGAGCGAG Antisense: TCAGAAGTCTCCAGAGCTTG	58	263	35
SOX9 (rt)	Sense: GGA GTGGAAGTTACTGACTGATG Antisense: AGGCGTTTTGCTTCGTCAATG	55	60	
**Hypertrophy and osteogenic markers**				
COL I	Sense: GGACACAATGGATTGCAAGG Antisense: TAACCACTGCTCCACTCTGG	54	461	32
COL X	Sense: CCCTTTTTGCTGCTAGTATCC Antisense: CTGTTGTCCAGGTTTTCCTGGCAC	54	468	25
OP	Sense: ACGCCGACCAAGGAAAACTC Antisense: GTCCATAAACCACACTATCACCTCG	51	483	35
ALP (rt)	Sense: TGGAGCTTCAGAAGCTCAACACCA Antisense: ATCTCGTTGTCTGAGTACCAGTCC	51	454	
IHH	Sense: GAGGAGTCCCTGCATTATGA Antisense: CAGGAAAATGAGCACATCGC	54	321	30
RUNX2	Sense: ACAGATGATGACACTGCCACC Antisense: CATAGTAGAGATATGGAGTGCTGC	55	324	35
**Internal control**				
EF1α	Sense: AGGTGATTATCCTGAACCATCC Antisense: AAAGGTGGATAGTCTGAGAAGC	54	234	25

rt: primer pairs, that have been used for real-time PCR only.

AGC = aggrecan core protein; ALP = alkaline phosphatase; COL = collagen; COMP = cartilage oligomeric matrix protein; EF1α = elongation factor 1α; FMD = fibromodulin; IHH = indian hedgehog; OP = osteopontin; RUNX2 = runt-related transcription factor 2; SOX9 = SRY (sex determining region Y) - box9.

For a more detailed mRNA expression profile of chondrogenic and hypertrophy associated genes, genetically-modified MSC aggregates were subjected to real-time quantitative PCR analyses. One microliter of each cDNA was used as template for amplification in a 50 μL reaction volume using BioTaq DNA Polymerase Taq (Bioline GmbH, Luckenwalde, Germany) and 50 pmol of gene-specific primers was used for COL II, SOX9, ALP and COL X as listed in Table 1. Real-time PCR conditions were as follows: 30 seconds at 94°C, 30 seconds at annealing temperature, 60 seconds at 72°C (see Table 1 for PCR conditions). Real-time PCR was performed with the DNA Engine Opticon system (MJ Research, Waltham, MA, USA) using SYBR Green (Biozym Scientific GmbH, Hessisch Oldendorf, Germany) as fluorescent dye allowing determination of the threshold cycle at which exponential amplification of PCR products begins. Specificities of amplicons were confirmed by melting curve analyses by gel electrophoresis of test PCR reactions. For quantification mRNA expression was normalized to the expression levels of the housekeeping gene EF1α and relative expression levels compared with values from undifferentiated monolayer MSCs are shown using the relative expression software tool (REST) [28]. Each PCR was performed in triplicate on three separate bone marrow preparations for each independent experiment.

### Statistical analysis

The data from the ELISA, WST1, ATP, GAG, DNA, and ALP content, cell surface area and RT-PCR analyses were expressed as mean values ± standard deviation (SD). Each experiment was performed in quadruplicate (n = 4) and repeated on at least three and up to six individual marrow preparations from different patients (m = 3 to 6), as indicated in the respective experiments. All numerical data were subjected to variance analysis (one or two factor analysis of variance) and statistical significance was determined by student's t-test, and level of *P *< 0.05 was considered significant.

## Results

### Transgene expression by aggregates of genetically modified MSCs

**Figure 1:**
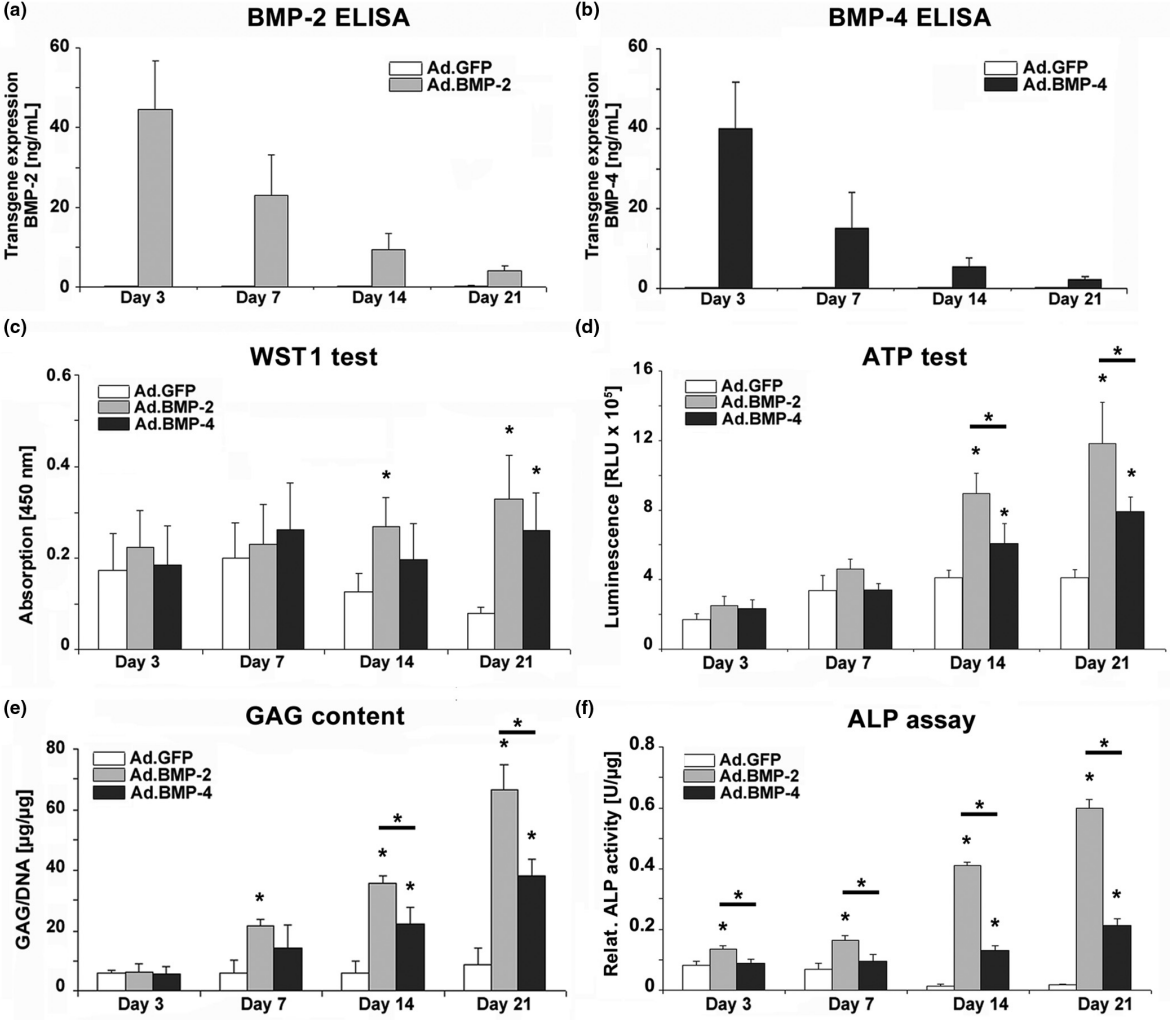
Transgene expression and biochemical composition of MSCs during 21 days of aggregate culture following BMP-2 and BMP-4 gene transfer. Primary MSCs were infected with Ad.BMP-2, Ad.BMP-4 or Ad.GFP at 5 × 10^2 ^vp/cell, seeded into aggregates and analyzed biochemically during a three-week time course. **(a, b) **Values represent levels of (a) BMP-2 and (b) BMP-4 transgene product expressed in ng/mL in the conditioned media over a 24-hour period at days 3, 7, 14 and 21. At the same time-points cell proliferation was quantified using the **(c) **WST1 and **(d) **ATP cell proliferation assay, **(e) **GAG content and **(f) **relative ALP activity normalized to DNA is shown. The data represent mean values ± standard deviation from four aggregates per condition and marrow preparation and was performed on five marrow preparations from different patients. Asterisks indicate values that are statistically different (*P *< 0.05) from marker gene vector-transduced control cultures or between samples. ALP = alkaline phosphatase; ATP = adenosine 5 triphosphate; Ad = adenoviral vector; BMP = bone morphogenetic protein; GAG = glycosaminoglycan; MSC = mesenchymal stem cell.

Consistent with previous findings [21], cultures infected with these doses of Ad.BMP-2 and Ad.BMP-4 generated approximately 30 to 60 ng/mL of gene product per 24 hours at day 3 post-infection (Figures 1a, b). The amount of each transgene product steadily decreased thereafter, and reached levels of about 3 to 6 ng/mL at day 21 (Figures 1a, b). Levels of BMP-2 and BMP-4 in media conditioned by Ad.GFP or Ad.Luc infected cultures were below 200 pg/mL (Figures 1a, b), equivalent to the levels observed in the naïve controls (data not shown).

### Cell proliferation, GAG content and ALP activity

As primary MSCs were shown to be capable of expressing the BMP-2 or the BMP-4 transgene in aggregate culture, we examined the effects of BMP-2 and BMP-4 gene delivery on cell proliferation using the WST1 cell proliferation assay. At day 3 and 7 of culture the cell proliferation rate in MSC aggregates was approximately equal in all groups tested (Figure 1c). BMP-2 and BMP-4 transduced MSC aggregates maintained their proliferation rate over 21 days while Ad.GFP cells (Figure 1c) and unmodified control cultures (not shown) decreased rate of proliferation (Figure 1c). The same pattern was observed using the ATP test, where sustained high cell proliferation rates were observed at day 14 and 21 in BMP-2- and BMP-4-modified aggregates compared with the controls, while at the same time points, levels in the BMP-2-modified aggregates were significantly elevated compared with the BMP-4 cultures (Figure 1d). To quantitatively compare extracellular matrix synthesis among treatment groups, GAG levels in the aggregates after 21 days in culture were determined (Figure 1e). All aggregates infected with Ad.BMP-2 or Ad.BMP-4 showed significantly increased GAG production relative to those receiving Ad.GFP (Figure 1e), Ad.Luc or untransduced aggregates (not shown), which showed no evidence of chondrogenesis. At days 14 and 21, significantly elevated levels of GAG synthesis in the BMP-2 compared with the BMP-4 transduced cultures became apparent (Figure 1e). Indicative of hypertrophic chondrocytes we analyzed ALP activity, which was found to be significantly elevated at all time points in the BMP-2-modified aggregates compared with the GFP controls and BMP-4 transduced cultures, whereas significantly higher values in the BMP-4 modified cultures compared with the GFP controls could only be resolved at day 14 and 21 (Figure 1f).

### Histological and immunohistochemical analyses of chondrogenesis

**Figure 2:**
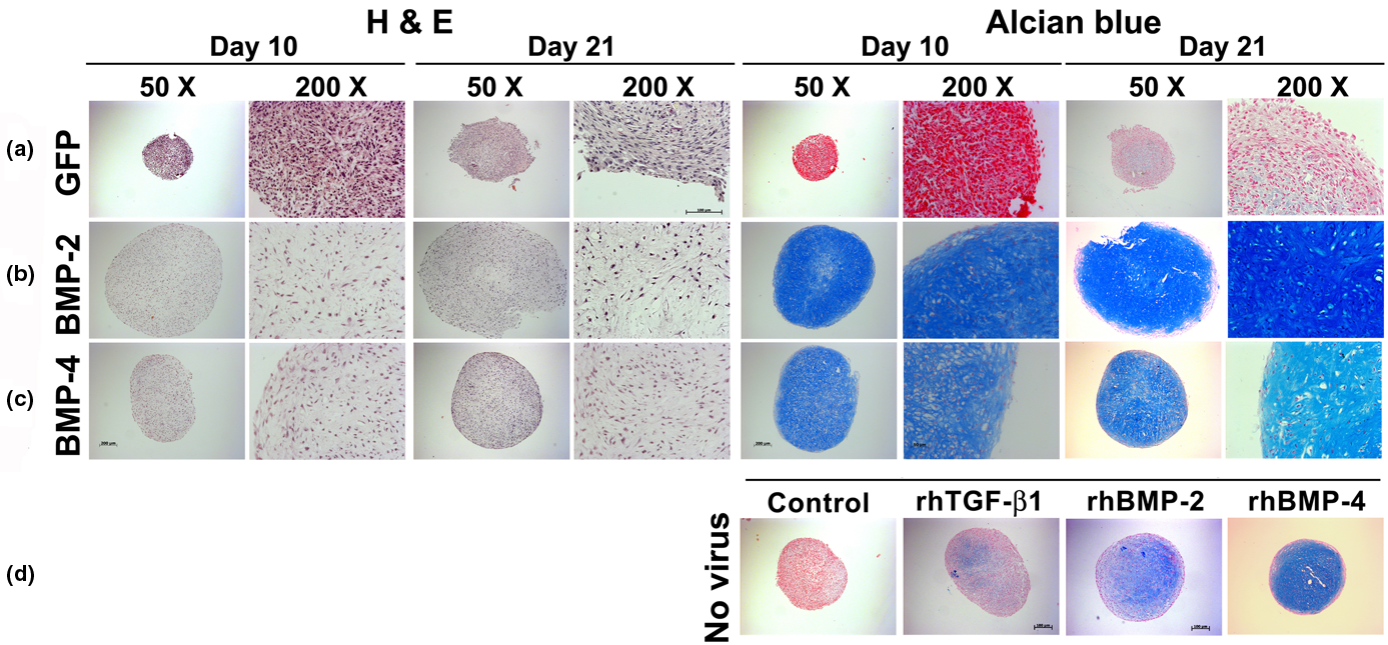
Histological appearance of MSC pellets after chondrogenic induction with BMP-2 or BMP-4 gene transfer. Monolayer cultures of MSCs were infected with **(a) **Ad.GFP, **(b) **Ad.BMP-2 or **(c) **Ad.BMP-4 at 5 × 10^2 ^vp/cell as indicated, seeded into aggregates 24 hours after infection and cultured in serum-free medium for 21 days. Representative sections after 10 and 21 days are shown. (Left panels) H&E staining for evaluation of cellularity and cell morphology. (Right panels) Alcian blue staining for detection of matrix proteoglycan. (a to c) Panels are reproduced at low (50×; bar = 200 μm) or high (200×; bar = 50 or 100 μm) magnification as indicated. **(d) **Comparative uninfected aggregate cultures after 21 days, that were maintained in the absence (control) or presence of recombinant human TGF-β 1 (10 ng/mL), or BMP-2 (25 ng/mL), or BMP-4 (25 ng/mL) protein as indicated. Panels are reproduced at low (50×; bar = 100 μm) magnification. Ad = adenoviral vector; BMP = bone morphogenetic protein; GFP = green fluorescent protein; H&E = hematoxylin and eosin; MSC = mesenchymal stem cell; TGF = transforming growth factor.

Transduction of MSCs with adenoviral vectors encoding BMP-2 (Figure 2b) or BMP-4 (Figure 2c) using viral doses sufficient to generate 30 to 60 ngs transgene product at day 3 induced a significant chondrogenic response in the respective aggregate cultures compared with the controls (Figure 2a), which were not chondrogenic. This was demonstrated by increased aggregate size and strong production of proteoglycans as indicated by metachromatic staining with alcian blue in the Ad.BMP-2 or Ad.BMP-4 transduced cultures (Figures 2b, c) compared with the Ad.GFP controls (Figure 2a). Interestingly, the phenotype of the Ad.BMP-4 (Figure 2c) infected aggregates appeared chondrogenic but less hypertrophic at day 10 and 21 compared with the Ad.BMP-2 cultures in that the BMP-2-modified cells were more rounded with greater cytoplasmic volume (Figure 2b).

**Figure 3:**
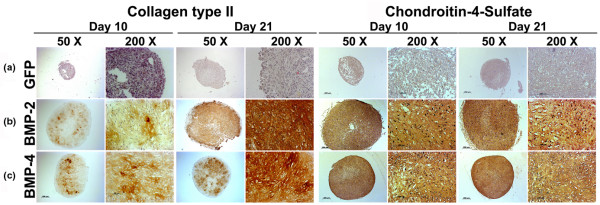
Immunohistochemical analyses for cartilage matrix proteins of MSC pellets after chondrogenic induction with BMP-2 or BMP-4 gene transfer. Monolayer cultures of MSCs were infected with **(a) **Ad.GFP, **(b) **Ad.BMP-2 or **(c) **Ad.BMP-4 at 5 × 10^2 ^vp/cell as indicated and placed into aggregate cultures. Immunohistochemical staining was performed on culture days 10 and 21 for collagen type II (left panels) and chondroitin-4-sulfate (right panels). Regions of positive immunostaining appear brown. Panels are reproduced at low (50×; bar = 200 μm) or high (200×; bar = 50 or 100 μm) magnification as indicated. Ad = adenoviral vector; BMP = bone morphogenetic protein; GFP = green fluorescent protein; MSC = mesenchymal stem cell.

Correspondingly, immunohistochemical analyses for COL II, the predominant collagen type in cartilage, and CS4, one of the monomers of the polysaccharide portion of proteoglycan, showed significantly enhanced production of these cartilage matrix proteins at days 10 and 21 of culture in the aggregates receiving Ad.BMP-2 (Figure 3b) or Ad.BMP-4 (Figure 3c) relative to the Ad.GFP (Figure 3a) controls.

Uninfected aggregates maintained in the presence of recombinant BMP-2, BMP-4, or TGF-β1 protein were also chondrogenic as evidenced by lacunae formation, positive staining for alcian blue (Figure 2d), COL II and CS4 (not shown), although the stage of chondrogenesis seemed less progressed compared with that in the aggregates genetically modified with BMP-2 or BMP-4 (Figures 2b, c) after 21 days, while control cultures where growth factor supplementation was absent were non-chondrogenic.

### Hypertrophic differentiation and apoptosis

**Figure 4:**
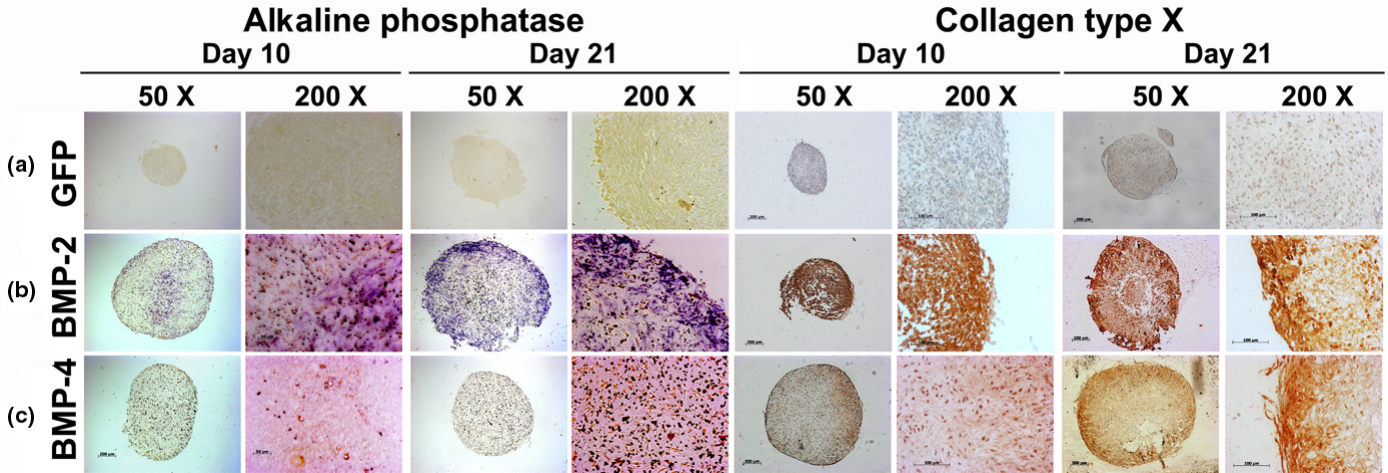
Histological and immunohistochemical analyses for hypertrophy of MSC pellets after chondrogenic induction with BMP-2 or BMP-4 gene transfer. Following genetic modification with **(a) **Ad.GFP, **(b) **Ad.BMP-2 or **(c) **Ad.BMP-4 aggregates after 10 and 21 days of culture stained for ALP (left panels) and collagen type X (right panels) are shown. Regions of positive immunostaining appear brown. Panels are reproduced at low (50×; bar = 200 μm) or high (200×; bar = 50 or 100 μm) magnification as indicated. Ad = adenoviral vector; ALP = alkaline phosphatase; BMP = bone morphogenetic protein; GFP = green fluorescent protein; MSC = mesenchymal stem cell.

We used staining for ALP and immunohistochemistry for COL X as markers for chondrocyte hypertrophy (Figure 4). No detectable ALP and only weak COL X immunostaining was seen in the control aggregates transduced with Ad.GFP (Figure 4a). ALP staining was primarily pericellular in the aggregates infected with Ad.BMP-4 (Figure 4c). In contrast, aggregates transduced with Ad.BMP-2 showed more abundant staining for ALP throughout the extracellular matrix at day 10 and was most extensive at day 21 of culture (Figure 4b). Correspondingly, immunohistochemical analyses of the Ad.BMP-2 infected aggregates revealed strong abundant staining for COL X in the aggregate matrix at day 10 and 21 of culture (Figure 4b). In the Ad.BMP-4-modified cultures COL X immunostaining of the matrix was strongly observed at day 21 in the aggregate matrix, while staining tended to be pericellular at day 10 of culture (Figure 4c); no significant differences were noted among the aggregates. Notably, the distribution pattern of the hypertrophy markers was somewhat heterogeneous in the aggregates, which we attribute to the rather inhomogeneous aggregate morphologies obtained during culture in v-bottom plates as opposed to more homogeneous aggregate morphologies seen after centrifugation and culture in 15 mL conical tubes [20].

**Figure 5:**
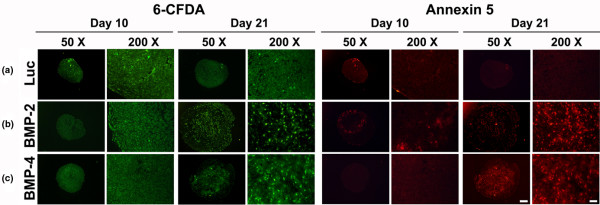
Analyses for cell viability and apoptosis within MSC pellets after chondrogenic induction with BMP-2 or BMP-4 gene transfer. Following genetic modification with **(a) **Ad.GFP, **(b) **Ad.BMP-2 or **(c) **Ad.BMP-4 aggregates were double-stained with 6-CFDA (left panels) and annexin 5-Cy3 (right panels) at day 10 and 21 of culture. Representative fluorescence microscopy images are shown. Note that living cells are stained green with 6-CFDA, late apoptotic cells red with annexin 5-Cy3, while early apoptotic cells stained for both 6-CFDA and annexin 5-Cy3. Panels are reproduced at low (50×; bar = 200 μm) or high (200×; bar = 50 μm) magnification as indicated. Ad = adenoviral vector; BMP = bone morphogenetic protein; CFDA = carboxyfluorescein diacetate; GFP = green fluorescent protein; MSC = mesenchymal stem cell.

Double fluorescence staining with Ann5-Cy3/6-CFDA allowed visualisation of Ann5 expressions. The high levels of green fluorescence found in BMP-modified (Figures 5b, c) and control groups (Figure 5a) revealed high viability of adenoviral infected MSCs in aggregate cultures after 10 and 21 days. At day 10, only very few cells in the Luc (Figure 5a) and BMP-2 (Figure 5b) and BMP-4 (Figure 5c) modified aggregates appeared to be annexin 5 positive. At day 21, the BMP-2 (Fig. 5B) and the BMP-4 (Fig. 5C) modified groups showed many Ann5-positive cells, as evidenced by red fluorescence, compared with the Ad.Luc transduced (Figure 5a) and untransduced (not shown) cultures where only very few such cells were seen.

A similar pattern of hypertrophy and apoptosis was observed in the untransduced control aggregates that were maintained in the presence or absence of recombinant BMP-2, BMP-4 or TGF-β1 protein (not shown).

### Comparison of BMP-2 and BMP-4 modified MSC aggregates with immature growth plate chondrocytes

In the different types of aggregates examined in Figures 2 to 5, different cell morphologies were apparent, especially with respect to incidence and extent of lacunae formation. Thus we were next interested to know if it was possible to distinguish the types of aggregates produced by measuring the sizes of the respective lacunae, approximated by simple histomorphometric cell surface area measurement on aggregate sections. For comparison, we first analyzed the sizes of the lacunae within different zones of growth plate cartilage obtained from a four-year-old child, from whom a sixth toe was removed. These measurements were compared with those of the lacunae found in the center and periphery of the different treatment groups of genetically modified aggregates.

**Figure 6:**
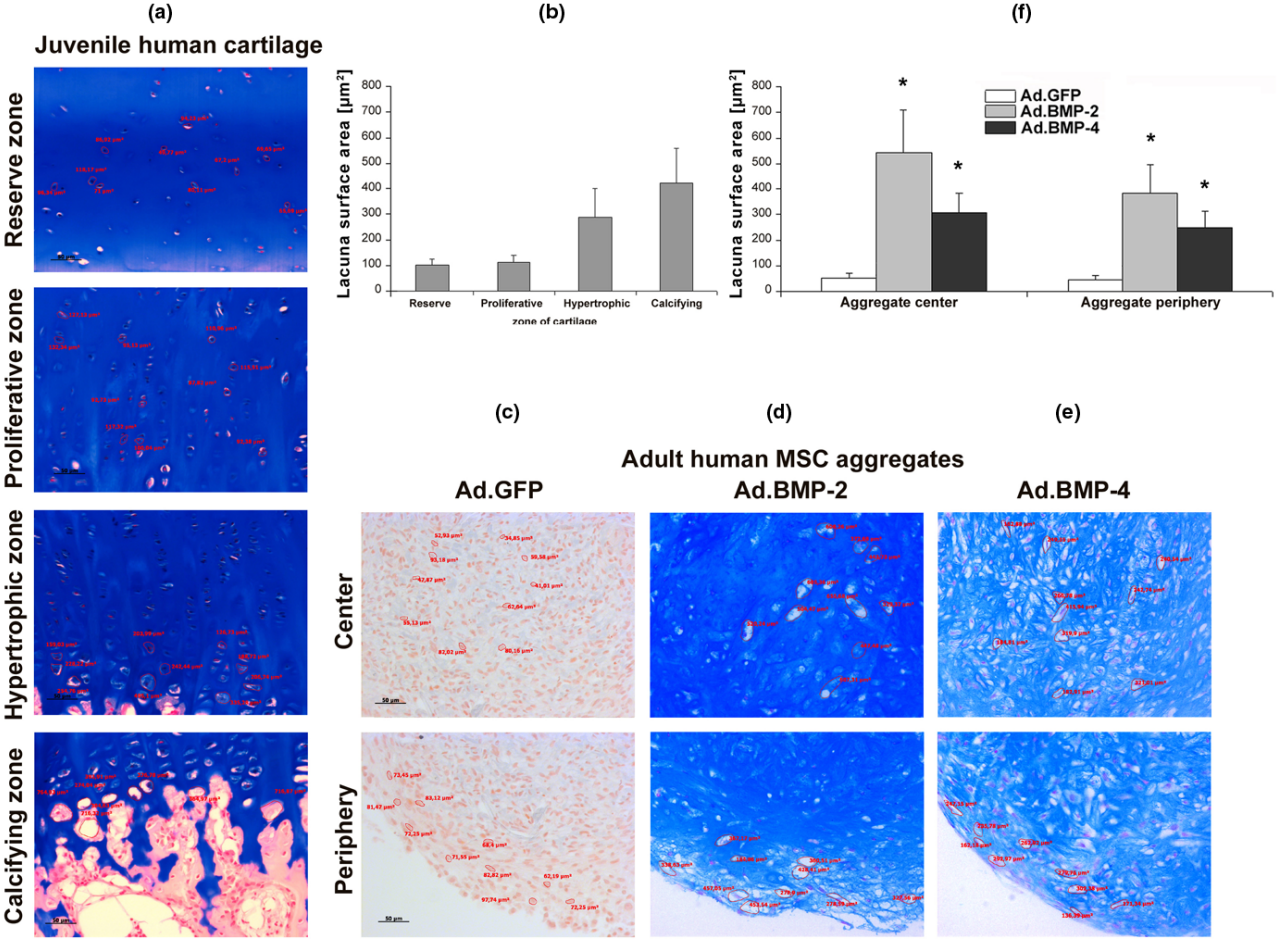
Analysis of hypertrophic cell morphology in MSC pellets after chondrogenic induction with BMP-2 or BMP-4 gene transfer in comparison to growth plate chondrocytes. Lacunar sizes were measured in the different zones of growth plate cartilage obtained from a four-year-old child, from which a sixth toe was removed. **(a) **The different cell morphologies and lacunar sizes in the reserve, proliferative, hypertrophic and calcifying zone of growth plate cartilage can be observed. **(b) **Measurements of the lacunar sizes in the respective zones are shown. Note that the mean values +/- SD represent analyses of size-measurements of 10 lacunae per zone, which were performed on three representative mid-sections per growth plate, and a total of four physes (one digit, two joints) were examined. **(c) **The GFP-modified aggregates showed no lacunae formation. **(d, e) **In contrast the BMP-2 and BMP-2 modified aggregates displayed a strong chondrogenic phenotype with formation of large lacunae in the (d) BMP-2 and (e) BMP-4 modified aggregates at day 21 of culture. **(f) **Analyses of lacunae surface areas in the center and periphery (outer 200 μm area) of the different aggregate types at day 21 of culture. The data represent mean values ± SD from four aggregates per condition and marrow preparation and was performed on six marrow preparations from different patients. Asterisks indicate values that are statistically different (*P *< 0.05) from marker gene vector-transduced control cultures. Thus both, the BMP-2 and the BMP-4 were significantly larger compared with the non-chondrogenic controls and displayed lacunar sizes comparable with those of the hypertrophic and calcifying zones of growth plate cartilage. Original magnification: 200×; scale bar = 50 μm. BMP = bone morphogenetic protein; GFP = green fluorescent protein; MSC = mesenchymal stem cell; SD = standard deviation.

As shown in Figure 6a, the reserve, proliferative, hypertrophic and calcifying zone of cartilage could be clearly separated by the proximity of the cells to the joint space and the bone respectively, alignment of the chondrocytes along the arcades of Benninghoff [29] and by the appearance of hypertrophic cells. Analyses of lacunae surface areas in the different growth plate zones revealed mean lacunae surface areas ± SD of 100.8 ± 25.8 μm^2 ^in the reserve zone, 113.3 ± 25.5 μm^2 ^in the proliferative zone, 288.5 ± 111.0 μm^2 ^in the hypertrophic zone and 421.8 ± 131.9 μm^2 ^in the calcifying zone of growth plate cartilage (Figure 6b). The mean values ± SD represent measurements of 10 lacunae per zone, which were performed on three representative mid-sections per growth plate. A total of four physes (1 digit, 2 joints) was examined.

In contrast the GFP-modified aggregates showed no lacuna formation, either in the center or in the periphery of the pellets (Figure 6c). However the BMP-2- and BMP-4-modified aggregates displayed a chondrogenic phenotype with lacunae formation throughout the aggregates (Figures 6d, e). Analyses of cell surface areas in the different aggregate types revealed a mean value of 60.6 ± 14.5 μm^2 ^in the center and 57.3 ± 12.4 μm^2 ^the periphery of the Ad.GFP transduced aggregates, which showed no lacunae formation, of 541.3 ± 166.3 μm^2 ^in the center and 386.1 ± 108.7 μm^2 ^the periphery of the Ad.BMP-2 transduced aggregates, and of 307.8 ± 75.6 μm^2 ^in the center and 248.7 ± 65.4 μm^2 ^the periphery of the Ad.BMP-4 transduced aggregates (Figure 6f). Thus lacunae formed in both the BMP-2 and BMP-4 transduced pellets and led to significantly larger cell surface areas compared with the non-chondrogenic controls. Nevertheless, the lacunae formed in the presence of BMP-2 were larger than those formed by BMP-4 and approximated the size of lacunae noted in the calcifying zone of the human growth plate. In contrast, the lacunae that formed in the presence of BMP-4 were closer in size to those of the hypertrophic zone (Figures 6e, f).

### Time course of chondrocytic and hypertrophic marker gene expression

**Figure 7:**
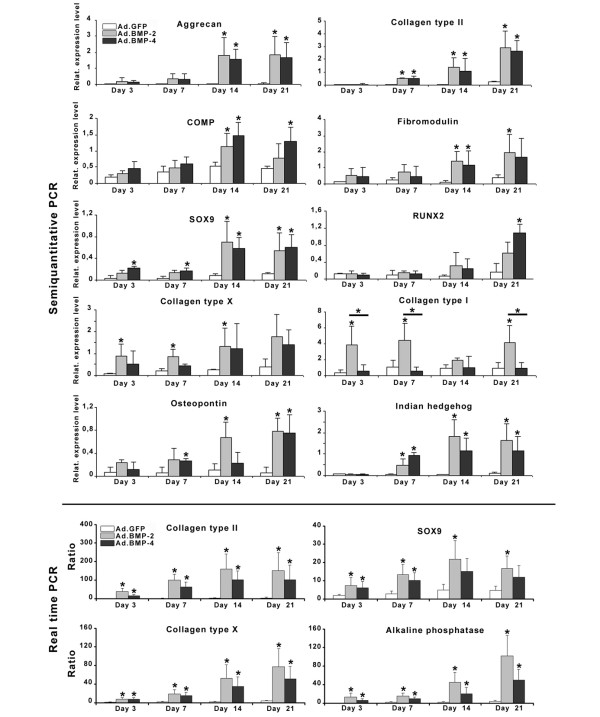
Profiles of temporal gene expression determined by semi-quantitative and real-time RT-PCR in MSC pellet cultures genetically modified with BMP-2 and BMP-4. Genes analyzed include collagen type (COL) II, aggrecan core protein (AGC), cartilage oligomeric matrix protein (COMP), fibromodulin (FMD), SRY (sex determining region Y) - box9 (SOX9), COL I, COL X, osteopontin (OP), indian hedgehog (IHH), runt-related transcription factor 2 (RUNX2) and alkaline phosphatase (ALP). Primer sequences and product sizes are listed in Table 1, with elongation factor 1α (EF1α) serving as housekeeping gene and internal control. For each marrow preparation/patient, treatment group and time point indicated RNA was extracted from 10 aggregates, and four patients were analyzed depending on group and time point. For the semi-quantitative RT-PCR analyses (upper panels), values are mean +/- SD raw data of optical band intensities of RT-PCR products between groups and time points (one band per patient), which were normalized using the EF1α reaction products. Values of the real-time RT-PCR analyses (lower panels) represent mean expression ratios +/- SD normalized to the expression levels of the housekeeping gene EF1α and relative to values from undifferentiated monolayer MSCs. Asterisks indicate values that are statistically different (*P *< 0.05) from marker gene vector-transduced control cultures or between samples. BMP = bone morphogenetic protein; MSC = mesenchymal stem cell; SD = standard deviation.

To examine further the effects of BMP-2 and BMP-4 gene delivery on hypertrophic differentiation, we analyzed the temporal expression profiles of genes associated with chondrocyte maturation and osteogenic differentiation using semi-quantitative and real-time RT-PCR (Figure 7). These genes included AGC, COL II, COMP, FMD, SOX9, RUNX2, COL X, COLI, ALP, OP and IHH. Consistent with preceding analyses [30], the aggregate cultures transduced with BMP-2 showed evidence of chondrogenic differentiation at the RNA level with upregulation of the chondrogenic markers AGC, COL II, FMD, COMP and SOX-9 over time, compared with the non-chondrogenic Ad.GFP controls where these markers were expressed only at low levels (Figure 7). Expression of these genes was upregulated to a similar degree in the BMP-4- and BMP-2-modified aggregates and marked differences between the BMP-2 and BMP-4 groups were not observed (Figure 7).

Evidence of chondrocyte hypertrophy at the mRNA level in the BMP-2- and BMP-4-modified aggregates was reflected by a subsequent upregulation of COL X and OP at day 3, IHH and ALP at day 7 and RUNX2 at day 14 compared with Ad.GFP controls (Figure 7). These results suggest that BMP-2 and BMP-4 gene transfer induced a significant chondrogenic and hypertrophic response in MSC aggregates on mRNA level over time.

## Discussion

We and others have shown previously that primary MSCs undergo chondrogenesis following genetic modification with Ad.BMP-2 or Ad.TGF-β1 in aggregate culture *in vitro *[30-32] or when transplanted into chondral defects *in vivo *[14]. In the present study we adapted the MSC aggregate culture system to determine whether adenoviral delivery of BMP-4 can lead to chondrogenesis of primary MSCs *in vitro*, and to evaluate the extent of hypertrophy compared with BMP-2-modified cultures.

Adenoviral delivery of BMP-4 led to reliable chondrogenesis in human MSC aggregate cultures in a fashion comparable with that noted when the same dose of the BMP-2 transgene was administered as shown by staining with alcian blue, COL II and CS4 and the quantitative GAG assay, indicating increased GAG levels at days 14 and 21 in the BMP-2-modified aggregates. Notably, chondrogenic differentiation induced by either transgene increase levels of metabolic activity and cell proliferation compared with controls as evidenced by the WST1 and ATP assays. Moreover, high levels of chondrocyte hypertrophy occurred in MSC pellet cultures modified with either BMP transgene, as assessed by lacunar size, and expression of ALP, COL X and Ann5, and was overall slightly more advanced in the BMP-2-modified cultures compared with the BMP-4 modified cultures reaching significance levels in the ALP assay at all time points. Notably, exact the lacunar size comparisons between growth plate tissues and *in vitro *cell pellets might be inaccurate (Figure 6) due to artifacts that may appear during fixation and processing of these different types of tissues.

The RT-PCR data are in general agreement with the biochemical and histological observations, showing high levels of chondrogenic mRNAs in aggregates after BMP-stimulation, such as AGC, COMP, COL II, SOX9 and FMD. Likewise, transcripts encoding the hypertrophy associated genes COL X, OP, ALP, RUNX2 and IHH were also strongly present in both types of BMP-modified aggregates compared with controls.

These observations are in broad agreement with our previous study using alginate cultures of the murine mesenchymal C3H10T1/2 cell line, stably transfected with BMP-2 or BMP-4 cDNAs, where similar differences in the pattern of chondrogenesis and hypertrophy were observed [33]. Although in this previous study the expression of osteogenic and hypertrophy markers were partly attributed to the presence of β-glycerophosphate, similar increases in hypertrophy associated genes were seen in the present study where β-glycerophosphate was absent. Our results are consistent with those reported by Mackay and colleagues [34] and Mueller and Tuan [35] who likewise showed that the addition of β-glycerophosphate is not necessary to obtain a hypertrophic chondrocyte phenotype.

Our study is also in agreement with studies of *in vitro *chondrogenesis with primary MSCs using recombinant proteins, where BMP-4 was identified as a strong inducer of chondrogenesis [36], which produced less hypertrophy compared with BMP-2 [37]. Correspondingly, *in vivo *implantation of BMP-4 into abdominal muscles of rats led to ectopic cartilage and bone formation when delivered as recombinant protein [38] or via genetically modified cells [39]. Notably, the latter study revealed differential effects on chondrogenesis and osteogenesis depending on the type of cell analyzed [39]. Our study is limited to the use of bone marrow-derived MSCs and other effects may be seen when different cells are employed. Orthotopic BMP-4 gene delivery via retrovirus transduction of muscle-derived stem cells was shown to improve cartilage repair in rat osteochondral defects [40] and also when it was administered via adenovirus to dedifferentiated chondrocytes in osteochondral defects in rabbits [41]. In both studies improved repair in the BMP-4-treated defects compared with non-chondrogenic controls at 12 or 24 weeks respectively was observed, but detailed analyses of hypertrophy and apoptosis have not been performed [40,41].

BMP-2 and BMP-4 have been implicated in embryogenesis and morphogenesis of various tissues and organs, where they regulate growth, differentiation, chemotaxis and apoptosis of a variety of cell types, including mesenchymal, epithelial, hematopoietic and neuronal cells [42]. Interestingly, in conditional knock-out experiments it has been found that a threshold level of BMP signaling is required for the onset of chondrogenesis, and hence some chondrogenic condensations failed to form in limbs deficient in both BMP-2 and BMP-4 [43]. However, in the condensations that do form, subsequent chondrogenic differentiation proceeds normally even in the absence of BMP-2 and BMP-4 [43]. In contrast, it was found that the loss of both BMP-2 and BMP-4 results in a severe impairment of osteogenesis. Deletion of BMP-4 alone did not impair osteogenesis or fracture repair, while deletion of BMP-2 alone did not impair osteogenesis but strongly prevented fracture repair [43-45]. This indicates that the presence of BMP-2 or BMP-4 is a prerequisite for osteoblastogenesis and these morphogens can apparently compensate for each other to a certain extent. However, they are less important for chondrogenesis [43-45].

During limb development, cartilage is gradually replaced by endochondral ossification, a process in which the chondrocytes mature, hypertrophy and express COL X with reduced production of COL II. Subsequently the cartilage becomes vascularized and infiltrated by osteoprogenitor cells, while the chondrocytes undergo apoptosis. The osteoprogenitor cells differentiate into osteoblasts, replacing the cartilage with mineralized bone; BMP-2 and BMP-4 are important regulators of these processes [46-48]. By using chondroprogenitor cells in high density, three-dimensional cultures these regulatory mechanisms can be partially recapitulated. Thus it is not surprising that studies on *in vitro *chondrogenesis using MSCs or chondrocytes incubated with members of the TGF-β superfamily reveal considerable hypertrophy and high levels of COL X expression. Although the use of COL X as a marker of chondrogenic hypertrophy in MSC-based systems has been questioned [13], it correlates well thus far to the existing *in vivo *data. For example, MSCs genetically modified to express BMP-2 display a significant level of tissue hypertrophy and osteophyte formation, when transplanted orthotopically to osteochondral defects [14] or ectopically [15,49] in small animal models. TGF-β1 has been shown to induce hypertrophic and osteometaplastic changes in the synovium of rabbit joints, when directly delivered by first-generation adenovirus [50]. Furthermore, implantation of chondrocytes genetically modified to express BMP-7 has been shown to generate good hyaline cartilage repair tissue after six weeks *in vivo*, but after one year the repair cartilage is no better than that of controls, with only 0 to 28% of the transplanted cells being detectable at that time point [51]. This is agreement with a recent large animal study in pigs, that showed good hyaline cartilage repair after six weeks, when chondral defects were filled with periosteum cells genetically modified with BMP-2, while at six months the hyaline repair tissue had almost completely vanished and was replaced by fibrocartilage [52]. These observations might be attributed to mechanisms of hypertrophic differentiation and subsequent apoptosis, although clarifying analyses *in vivo *have not been conducted thus far. However, the presence of Ann5-positive cells in our hypertrophic aggregates modified with BMP-2 or BMP-4 *in vitro *correspond with these data.

Our data suggest that the degree of hypertrophic differentiation can be modulated by the choice of morphogenetic stimulus, while still maintaining efficient chondrogenesis. This permits cautious optimism that it may prove possible ultimately to achieve effective regeneration of articular cartilage in the absence of hypertrophic differentiation. Hypertrophic differentiation of neo-cartilage tissue with subsequent apoptosis development is certainly an undesired effect in cartilage defects *in vivo*, because this would lead to loss of the transplanted repair cells with subsequent matrix degradation. However, the relevance of chondrogenic hypertrophy and apoptosis of human MSCs induced by TGF-β superfamily members for cartilage repair *in vivo *has to be considered still unclear to this end, because this study is limited by its *in vitro *nature. Therefore, clarifying *in vivo *experiments are necessary before such factors can be recommended for further clinical use.

## Conclusions

Adenoviral BMP-4 gene transfer efficiently induces the chondrogenic differentiation of human primary MSCs as effectively as BMP-2 gene transfer. However, both transgenes induced high levels of chondrocyte hypertrophy after three weeks of *in vitro *culture. It remains to be seen, whether it may be possible to develop methods for allowing robust chondrogenesis while preventing hypertrophic differentiation using different genes or proteins, which would presumably improve the outcome of cell-based approaches to cartilage repair *in vivo*.

## Abbreviations

AGC: aggrecan core protein; ALP: alkaline phosphatase; Ann: Annexin; ATP: adenosine 5 triphosphate; Ad: adenoviral vector; BMP: bone morphogenetic protein; BSA: bovine serum albumin; CFDA: carboxyfluorescein diacetate; COL: collagen; CS: chondroitin sulphate; COMP: cartilage oligomeric matrix protein; DMEM: Dulbecco's modified eagle media; EF1α: elongation factor 1α; ELISA: enzyme linked immunosorbent assay; FBS: fetal bovine serum; FGF: fibroblast growth factor; FMD: fibromodulin; GAG: glycosaminoglycan; GFP: green fluorescent protein; H&E: hematoxylin and eosin; Ig: immunoglobulin; IHH: indian hedgehog; Luc: luciferase; MSC: mesenchymal stem cell; OP: osteopontin; PBS: phosphate-buffered saline; PCR: polymerase chain reaction; RUNX2: runt-related transcription factor 2; SD: standard deviation; SOX9: SRY (sex determining region Y) - box9; TBS: Tris-buffered saline; TGF: transforming growth factor.

## Competing interests

The authors declare that they have no competing interests.

## Authors' contributions

All authors have read and approved the manuscript and contributed to the study design, data analysis, interpretation of data and drafting and revision of the manuscript. The data have been generated by AFS, BP, MK, SCG, and a data review committee (AFS, CH, SCG, AR, UN, JE and CHE) analysed the data.
